# Effects of matcha tea extract on cell viability and peroxisome proliferator-activated receptor γ expression on T47D breast cancer cells

**DOI:** 10.1007/s00404-021-06381-4

**Published:** 2022-01-25

**Authors:** Simon Keckstein, Constantin Tilgener, Udo Jeschke, Simone Hofmann, Theresa Vilsmaier, Till Kaltofen, Helene Heidegger, Falk Batz, Sven Mahner, Lennard Schröder

**Affiliations:** 1 Department of Obstetrics and Gynecology, University Hospital, LMU Munich, Marchioninistr. 15, 81377 Munich, Germany; 2 Department of Urology and Transplant Surgery, Klinikum Stuttgart, Kriegsbergstraße. 60, 70174 Stuttgart, Germany; 3 Department of Obstetrics and Gynecology, University Hospital Augsburg, Stenglinstr. 2, 86156 Augsburg, Germany

**Keywords:** Matcha tea extract, MTE, T47D, PPARγ, WST-1, PCR, Western blot

## Abstract

**Purpose:**

In the following work, we investigated the nuclear peroxisome proliferator-activated receptor gamma (PPARγ)-dependent proliferation behavior of breast cancer cells after stimulation with matcha green tea extract (MTE).

**Methods:**

T47D cells were stimulated with MTE at concentrations of 5, 10 and 50 µg/ml. Cell viability was assessed using a WST-1 assay after an incubation time of 72 h. PPARγ expression was quantified at the gene level by real-time polymerase chain reaction (PCR). A western blot (WB) was carried out for the qualitative assessment of the expression behavior of on a protein level.

**Results:**

The WST-1 test showed a significant inhibition of viability in T47D cells after 72 h at 5, 10 and 50 µg/ml. The PCR showed an overexpression of PPARγ in T47D cells in all concentrations. At the concentration of 50 µg/ml the expression was significantly increased (*p* < 0.05). The WB demonstrated a significant quantitative increase of PPARγ at protein level with MTE concentrations of 10 and 50 µg/ml. In addition, there was a negative correlation between the overexpression of PPAR γ and the inhibition of proliferation.

**Conclusion:**

MTE decreases the cell viability of T47D cells and furthermore leads to an overexpression of PPARγ on protein and mRNA level.

## Introduction

Breast cancer is the most frequent cancer in women worldwide. Increases in incidence over the last decades have been linked to the implementation of screening programs [1]. In 2018, around 2.1 million new cases were diagnosed worldwide and 626,679 women died of breast cancer [2]. Modern multimodal therapeutic concepts have enabled cure rates of 70–80% [3]. To achieve further improvements, the investigation of mechanisms and possible substances that may inhibit cancer proliferation and invasion remains of interest. Second only to water, tea is the most commonly consumed beverage worldwide [4] and in western countries its popularity is increasing further. Like black and green tea (GT), matcha tea (MT) is obtained from the leaves of the tea plant Camellia sinensis. While with GT the tea leaves are removed from the tea before consumption, with MT they are ground into a fine powder, dissolved in boiling water and ingested in their entirety; therefore, the intake of various substances contained in the leaves is much higher [4–6]. MT contains polyphenols, including the subdivisions of flavonoids, flavanols and catechins. Up to 30% of the dry mass of fresh tea leaves is made up of phenolic compounds, 90% of which are catechins. Of the catechins found in MT, epigallocatechin gallate (epigallocatechin-3-gallate, EGCG) is most abundant [7]. Other catechins such as epigallocatechin (EGC), epicatechin gallate (ECG) and epicatechin (EC) only make up 10%. The remaining dry mass components in tea leaves are proteins (15%), amino acids (4%), caffeine (4%), raw fibers (26%), lipids (7%), other carbohydrates (7%), pigments, such as chlorophyll and carotenoids (2%) and minerals (5%) [8]. 100 ml of GT contains approximately 20–100 mg of EGCG [9]. The main limitation for broad therapeutic application of polyphenols is that after oral intake and metabolism only as little as 0.024% are bioavailable [10, 11]. Results of pharmacokinetic studies concluded that after ingestion, EGCG concentrations in human serum may reach no more than the high nanomolar range [12]. The concentrations we used in vitro in the study presented here are deliberately lower than those used in previous experiments, but still substantially higher than serum concentrations measured in vivo after consumption and metabolism by humans. EGCG has been extensively investigated for its tumor inhibitory effects in breast and prostate cancer [13–16]. Different mechanisms by which EGCG can influence the cell cycle and proliferation have been described, such as the inhibition of matrix metalloproteinases [17], Wnt signaling [18], methylation [19] and induction of peroxisome proliferator-activated receptors (PPAR) [20–22]. The PPAR family of essential nuclear receptors includes three subtypes (α, β/δ, and γ) that bind directly to certain DNA regions and regulate the expression of target genes [23]. Previous research suggests that PPARγ has a pivotal role in the pathophysiology of multiple diseases and possibly their treatment. For instance, PPARγ is one of the major transcription factors activated by thiazolidinediones [24]. Research on PPARγ in tumor cells has yielded divergent results; in some studies it acted as a tumor suppressor, but others reported growth-promoting effects [25–28]. Investigations regarding breast cancer report mostly tumor suppressing effects [27, 29, 30]. With current data supporting that Camellia sinensis has a tumor suppressing effect on breast cancer cells, we wanted to elucidate a possible involvement of PPARγ. With diverging results of a possible cell inhibitory effect of EGCG on T47D [31, 32], we, therefore, investigated the effect of MTE in different concentrations on T47D breast cancer cells. Cell proliferation was analyzed using the WST-1 array. PPARγ expression was measured via real-time PCR and PPARγ protein levels were determined via western blot.

## Materials and methods

### Cell cultivation and cell stimulation

To mimic ductal breast carcinoma, the T47D cell line was used. Cells were grown on 80% monolayer in a cell culture bottle and cultivated in Dulbecco's Modified Eagle Medium (DMEM; 3.7 g/L NaHCO3, 4.5 g/L d-glucose, 1.028 g/L stable glutamine, and sodium pyruvate; Biochrom, Berlin, Germany). Then 10% heat-inactivated fetal calf serum (FCS; Biochrom) was added to the medium and incubated with atmospheric concentrations of CO_2_ of 5% at 37 °C. For further use they were trypsinized and counted.

### Preparation of the matcha tea extract (MTE)

Matcha tea was purchased commercially (Houjo Matcha Tea, harvested in Hoshino, Yame prefecture, Japan). For each test, the tea extract preparation was done as described in each test below.

### WST-1 assay

T47D cells were cultivated in a 96-well plate at a density of 10,000 cells per well in 50 µl DMEM with 10% FCS. After 4 h, the medium was replaced with DMEM without FCS. FCS is known to contain substances affecting cell proliferation and maintenance such as growth factors, hormones, vitamins, and transport proteins. By using DMEM without FCS, a possible effect of FCS components on cell proliferation was reduced [33, 34]. Cells were incubated for a further 12 h. 27.1 mg tea extract were dissolved in 100 µl pure ethanol and diluted 1:1000 with DMEM without FCS. A control solution was prepared in the same way without adding MTE. Afterwards, different amounts of the MTE solution and DMEM without FCS were added to achieve the desired concentrations (5, 10 and 50 µg/ml), resulting in a total of 100 µl per well. For the control group, instead of the MTE solution, the control solution and DMEM without FCS were added to each well. The wells were then incubated for 72 h. After incubation, the WST-1 reagent (water soluble tetrazolium, 4-[3-(4-iodophenyl)-2-(4-nitrophenyl)-2H-5-tetrazolio]-1,3-benzene disulfonate; Sigma-Aldrich, St. Louis, MO, USA) was added to detect the activity of mitochondrial succinate dehydrogenase cleaving the tetrazolium salt to formazan. Cell viability was measured after incubating for 30 min with the use of a multi-well spectrophotometer (wavelength: 420–480 nm). Three independent measurements with three technical replicates were performed.

### PCR

T47D cells were incubated on a 12-well plate at a density of 500,000 cells per well for 4 h with 500 µl DMEM with 10% FCS. Then the medium was replaced with 500 µl DMEM without FCS and the cells were incubated for a further 12 h. 27.1 mg tea extract were dissolved in 100 µl pure ethanol and diluted 1:1000 with DMEM without FCS. A control solution was prepared in the same way without adding MTE. Afterwards, different amounts of the MTE solution and DMEM without FCS were added to achieve the desired concentrations (5, 10 and 50 µg/ml), resulting in a total of 500 µl per well. For the control group, instead of the MTE solution, the control solution and DMEM without FCS were added to each well. The wells were then incubated for two hours. This time was selected because changes in mRNA levels are expected within hours after stimulation and the half-life of mRNA also being in the range of hours. Longer incubation times may, therefore, be unsuitable for detection of short-term changes in mRNA levels. The excess liquid was removed. The wells were washed with phosphate-buffered saline (PBS) and RA-1 buffer (Macherey–Nagel, Düren, Germany) was added for cell lysis. For RNA isolation, the NucleoSpinRNAII (Macherey–Nagel, Düren, Germany) was used. For the reverse transcription of RNA, the High-Capacity cDNA Reverse Transcription Kit (Thermo Fisher Scientific, Waltham, MA, USA) was used. 10 ng of RNA were added. The temperature protocol phases were 10 min at 25 °C, 2 h at 37 °C, 5 s at 85 °C and stopped with a cooling phase at 4 °C. For the TaqMan^®^ PCR, 96-well plates with 20 µl well volume were used. Each well was filled with 10 µl of TaqMan^®^ Universal PCR Master Mix 2X (Thermo Fisher Scientific), 8 µl of distilled water treated with 0.1% diethyl pyrocarbonate (DEPC), 1 µl of TaqMan^®^ Gene Expression Assay 20X (Thermo Fisher Scientific; target: ACTB, assay ID Hs99999903_m1; target: PPARG, assay ID Hs01115513_m1; sequences of the primers not revealed by manufacturer), and 1 µl of cDNA sample. The PCR assay was performed using the ABI Prism 7500 Fast (Thermo Fisher Scientific). Thermal cycling was started for 20 s at 95 °C and was followed by 40 cycles of amplification at 95 °C for 3 s, and 60 °C for 30 s. To analyze the results, the comparative 2^−ΔΔCT^ method was used [35]. As endogenous control for the ΔCT-values, β-actin was used. Three independent measurements with two technical replicates were performed.

### Western blot

**Fig. 1:**
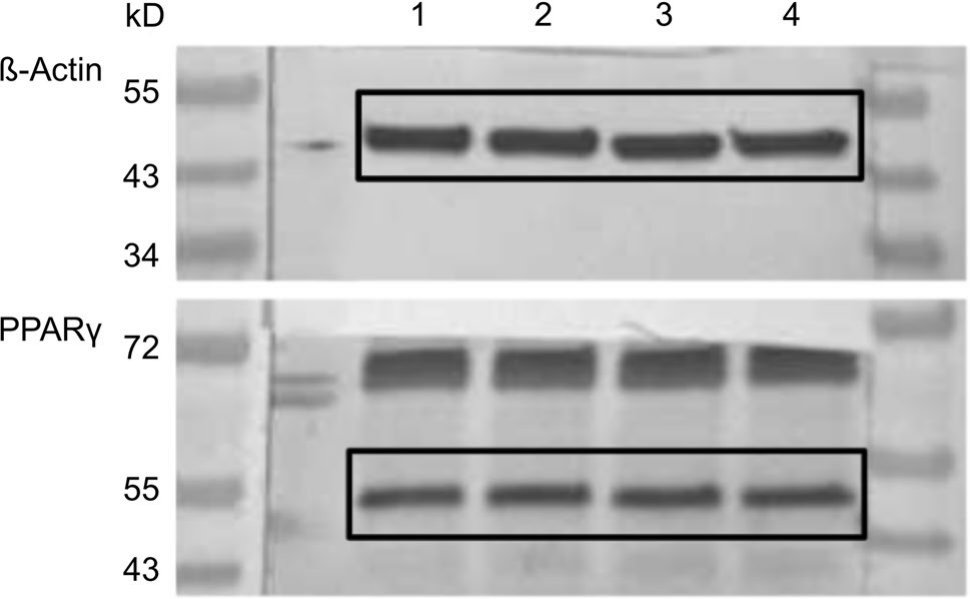
Example of western blot membranes after stimulation with different concentrations of MTE or the control group and incubation with β-actin and PPARγ antibodies. The respective bands are numbered [(1) 0 µg/ml; (2) 5 µg/ml; (3) 10 µg/ml; (4) 50 µg/ml] and marked with black boxes.

T47D cells were cultivated in a 12-well plate at a density of 500,000 cells per well in 1000 µl DMEM with 10% FCS. After 4 h, the medium was replaced with 1000 µl DMEM without FCS and the cells were incubated for a further 12 h. 20 mg tea extract were dissolved in 100 µl pure ethanol and diluted 1:1000 with DMEM without FCS. The dilution for the control cells was prepared in the same way without the addition of MTE. Afterwards, different amounts of the MTE solution and DMEM without FCS were added to achieve the desired concentrations (5, 10 and 50 µg/ml), resulting in a total of 1000 µl per well. For the control group, instead of the MTE solution, 250 µl control solution and 750 µl DMEM without FCS were added to each well. Cells were incubated for 48 h, then washed with phosphate-buffered saline (PBS). For cell lysis, 200 µl of a buffer solution consisting of a 1:100 of protease inhibitor (Sigma-Aldrich, St. Louis, MO, USA) in RIPA buffer (radioimmunoprecipitation assay buffer; Sigma-Aldrich) was added to each well before incubating for 30 min at 4 °C. After centrifuging the lysates, a Bradford protein assay of the supernatant was performed. With western blotting, the proteins were separated depending on their molecular weight with the use of SDS-PAGE and transferred onto a polyvinylidene fluoride (PVDF) membrane (Merck Millipore, Darmstadt, Germany). After blocking the PVDF membrane for 1 h in a receptacle containing a casein solution 1× (Vector Laboratories, Burlingame, CA, USA) to prevent a nonspecific binding of the antibodies, the primary antibodies anti-β-actin (clone AC-15, mouse IgG; Sigma-Aldrich Co., St. Louis, Missouri, USA) and anti-PPARγ (polyclonal IgG, rabbit, Abcam, Cambridge, UK) were diluted in a 1×casein solution and afterwards laid onto the membrane for 16 h at 4 °C. After washing the membranes with tris-buffered saline (TBST), membranes were incubated with biotinylated anti-rabbit IgG antibody and ABC-AmP reagent (VECTASTAIN ABC-AmP Kit for rabbit IgG, Vector Laboratories) according to the manufacturer’s protocol. Specific bands on the membrane were visualized using the BCIP/NBT chromogenic substrate (Vectastain ABC-AmP Kit, Vector Laboratories), detected with the Bio-Rad Universal Hood II (Bio-Rad Laboratories, Hercules, CA, USA) and the specific color densities of the bands were quantified with the Bio-Rad Quantity One software (Bio-Rad Laboratories) (Fig. 1). For statistical evaluation, the color intensity of PPARγ bands clustered pixels were set in relation to the β-actin bands clustered pixels. The western blots were repeated independently five times.

### Statistical analysis

The statistical programming environment R, version 4.0.2 [36], was used for processing and statistical analysis of the collected data. Findings with *p* values < 0.05 were considered significant. Shapiro–Wilk tests were used to check normality of distributions. Adapted to the specific design of experiment we applied different statistical tests to compare stimulated and control samples. The paired *t* test was used for the WST-1 and PCR assay. To account for repeated measurements in several Western-blot analyses we fitted a linear random effects model.

## Results

### WST-1 proliferation assay

**Fig. 2:**
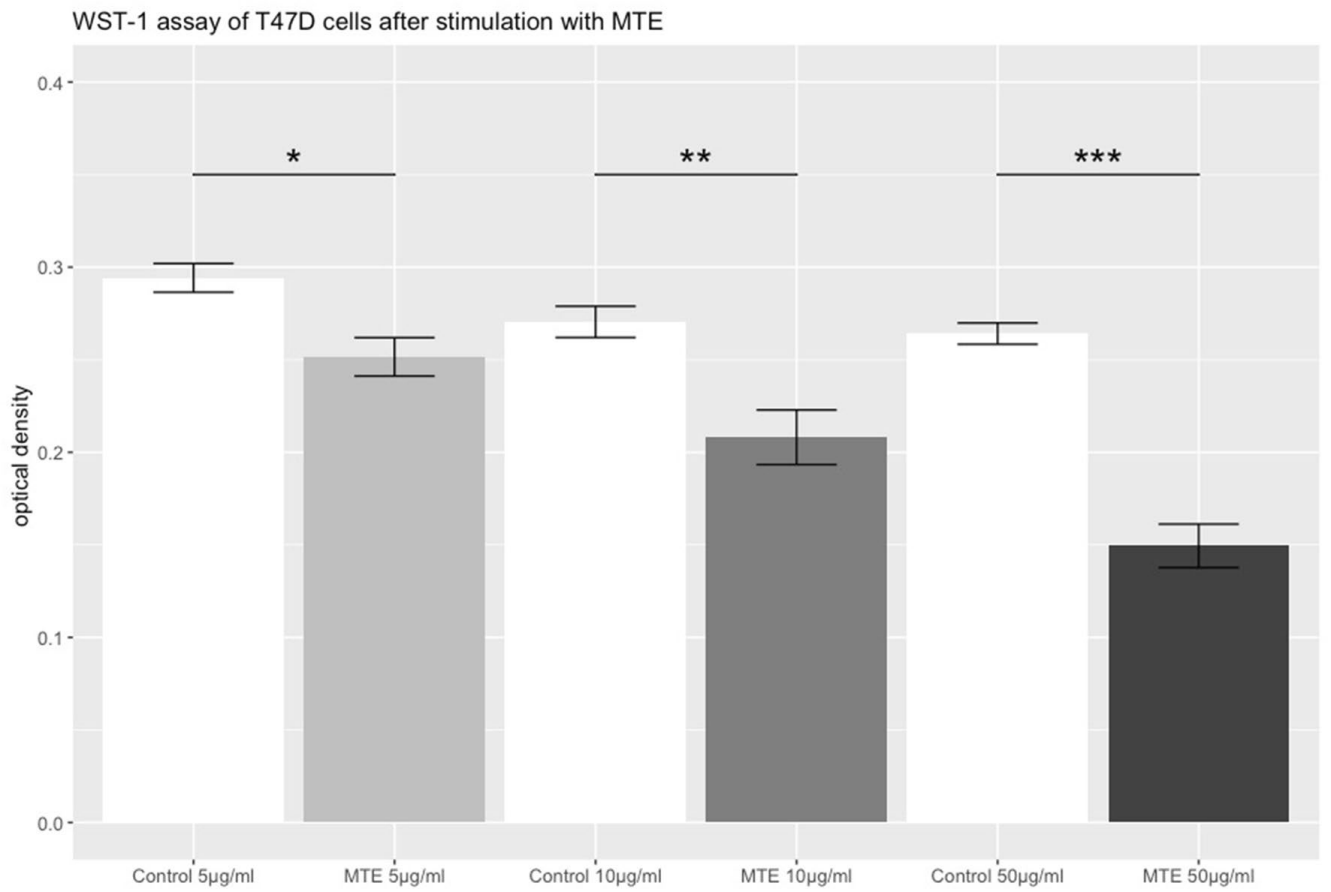
WST-1 assay of T47D cells stimulated with MTE. The grey bars represent the optical density of T47D cells after the incubation with different concentrations of MTE (5, 10 and 50 µg/ml) for 72 h. The white bars represent the control group. The top of each bar represents the mean ± standard error (SE). MTE induced a significant reduction of cell proliferation at every concentration. Significant results are linked and marked with asterisks (*p* < 0.05*, *p* < 0.01**, *p* < 0.001***)

To detect changes in T47D cellular viability after incubation, a WST-1 proliferation assay was performed. A significant inhibitory effect in cell viability was observed (*p* = 0.017) already at the concentration of 5 µg/ml MTE. As the concentrations of MTE increased, higher levels of significance where observed (10 µg/ml MTE: *p* = 0.002; 50 µg/ml MTE: *p* < 0.001). Results are shown in Fig. 2.

### PCR for PPARγ expression on mRNA level

**Fig. 3:**
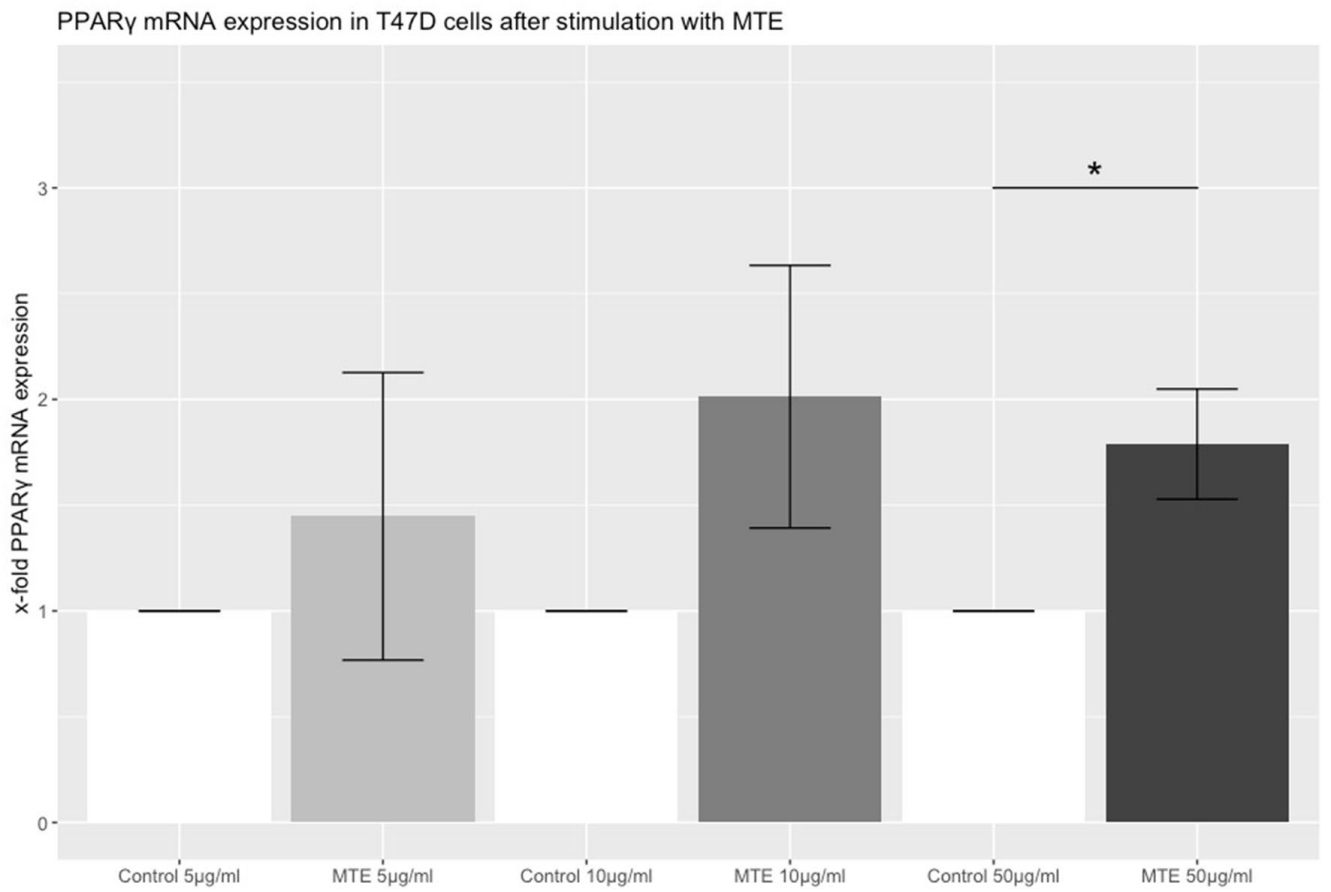
The bar chart represents the relative PPARγ expression on mRNA level in T47D cells after incubation with three different concentrations of MTE (5, 10 and 50 µg/ml) or the control solution for 2 h. mRNA levels were detected via TaqMan^®^ real-time PCR. The values on the *y* axis are show ratios of stimulated and control expression levels. The top of each bar represents the mean ± SE. Statistical significance was achieved at the highest concentration of MTE (*p* = 0.029) and is marked with one asterisks

To detect changes of PPARγ mRNA expression after incubation of T47D cells with different MTE concentration, TaqMan^®^ PCR was performed. To analyze the results, the comparative 2^−ΔΔCq^ method was used. The *x* fold expression of mRNA in comparison to the control increased in a dose-dependent manner. Whereas at the concentrations of 5 µg/ml and 10 µg/ml mRNA expression of PPARγ was numerically higher than control, this increase was only statistically significant at the highest concentration of 50 µg/ml MTE (5 µg/ml MTE: *p* = 0.539; 10 µg/ml MTE: *p* = 0.163; 50 µg/ml MTE: *p* = 0.029). Results are shown in Fig. 3.

### Western blot for PPARγ on protein level

**Fig. 4:**
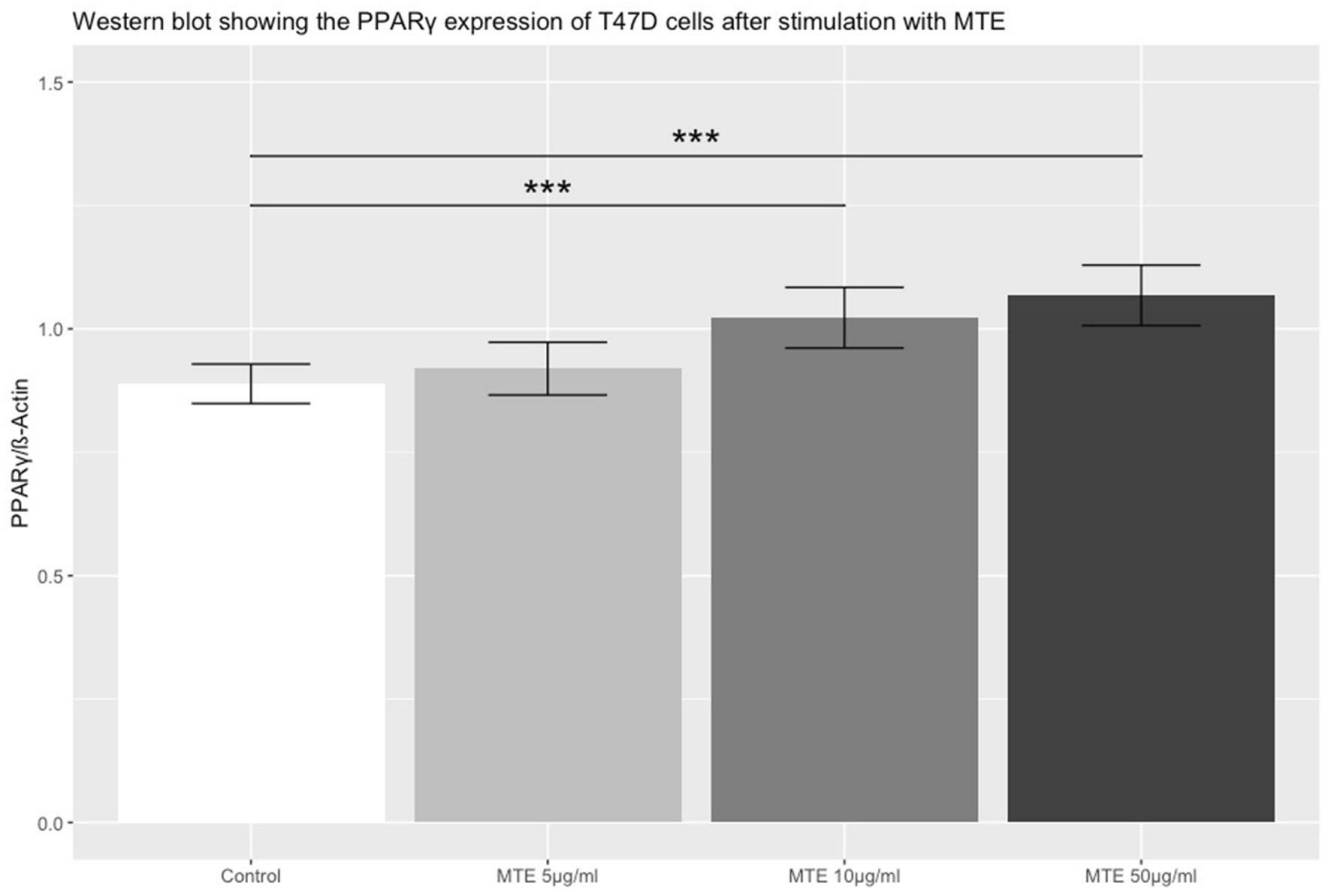
The bar chart shows the PPARγ protein expression in T47D cells after stimulation with different concentrations of MTE compared to control. The top of each bar represents the mean ± SE. MTE induced a significant upregulation of PPARγ expression on protein level at MTE concentrations of 10 µg/ml and 50 µg/ml. Significant differences between groups are linked and marked with asterisks (*p* < 0.001***)

To investigate PPARγ expression on a protein level, a western blot was applied. At all MTE concentrations PPARγ expression increased in a dose-dependent manner in comparison to the control cells. PPARγ expression was significantly elevated at MTE concentrations of 10 and 50 µg/ml (5 µg/ml MTE: *p* = 0.315; 10 µg/ml MTE: *p* < 0.001; 50 µg/ml MTE: *p* < 0.001). Results are shown in Fig. 4.

## Discussion

To our knowledge, this is the first study investigating the effect of MTE on PPARγ expression in T47D breast cancer cells. Natural substances have a long history in the prevention and treatment of neoplasms [37]. Green tea and matcha tea—with their chemical compounds including vitamins, phenolic acids, quercetin and catechins—have been associated with health benefits such as anti-inflammatory and anti-carcinogenic effects [38]. EGCG is the most abundant catechin in matcha tea. Its potent growth inhibitory effects have been demonstrated in various cell lines [39, 40]. A possible anti-carcinogenic effect of EGCG on the estrogen receptor positive, HER2 negative, T47D cell line has been investigated in the past yielding controversial results. While Moradzadeh et al. were able to demonstrate decreased cell viability after treatment with EGCG [31], Zeng et al. could not find an anti-carcinogenic effect of EGCG on T47D cells alone [32]. We therefore wanted to investigate the potential antiproliferative effects and effects on PPARγ expression of matcha tea extract on T47D cells. In this study, we used an extract instead of its individual active compounds to evaluate the cumulative and synergistic anti-carcinogenic effect of all active compounds in MT. In previous studies, we could demonstrate anti-carcinogenic effects on both ER/PR receptor-positive and -negative breast cancer cells using GT, MT, EGCG and quercetin [40]. Our hypothesis in this trial was that PPARγ induction may be responsible for some of the observed anti-carcinogenic effects.

It has been proposed that MTT/MTS-based assays may underestimate an antiproliferative effect of EGCG. EGCG has been described to induce an increased activity of the mitochondrial dehydrogenase and furthermore has an intrinsic potential to reduce MTT and MTS and therefore increase the formation of formazan [41–43]. The significant reduction in cell growth by all used concentrations underlines the anti-carcinogenic effect of matcha tea extract on T47D cells in vitro. The main limitations of the methods used in this in vitro study are the use of concentrations not achievable in vivo by oral ingestion of MT and that MTE has not been subjected to prior digestion, intestinal absorption, and first pass metabolism. In 1997, Nagakawa and Miyazawa demonstrated that due to the complexity of digestion, absorption and metabolism, the maximum serum plasma concentration of EGCG is only 0.024% of the amount ingested [10]. In a study by Yang et al. 18 individuals consumed 3 g of decaffeinated GT (EGCG content: 73 mg/g) which resulted in an EGCG plasma concentration of no more than 326 ng/ml. Whereas higher amounts of GT consumption (4.5 g) did not lead to increased plasma concentrations, an increase from 1.5 to 3 g resulted in a triple EGCG plasma concentration [44]. The lowest concentration used in our study was 5 µg/ml, equal to 5000 ng/ml MTE. Weiss et al. described concentrations of EGCG after extraction with water of 0.32 mg per gram of MT [45]. Transferring these findings, the amount of EGCG at the given concentrations of 5, 10 and 50 µg/ml are higher than those that are achievable by oral ingestion of MT. EGCG metabolism such as methylation, glucuronidation, sulfation, and oxidative degradation further weakens its efficacy. For example, the catechol-*O*-methyltransferase (COMT) reduces the amount of EGCG available. Carriers of the lesser potent COMT-L allele benefit more from catechin activity than carriers of the highly potent COMT-H allele [46]. To overcome low bioavailability, several different approaches have been investigated. A simple method is the application of EGCG capsules where dosages of 800 mg resulted in plasma levels of up to 439 ng/ml [47]. Approaches that are more complex include modification of the EGCG molecule. For example, it has been demonstrated that the addition of peracetate protections to EGCG hydroxy groups leads to higher bioavailability and higher concentrations in breast cancer cells [48]. Moreover, recently several different advanced nanovehicle-enabled delivery systems of EGCG have been tested in various cell and animal models that demonstrate better bioavailability and promising results in the context of cancer therapy [49]. Due to the facts described above, it becomes clear that although relevant results regarding PPARγ induction were demonstrated, this in vitro study cannot simulate the conditions encountered in human physiology. Animal studies might provide more insight.

Despite the methodological limitations mentioned above, the strength of our study lies in the fact that while many in-vitro studies have until now extensively investigated steroid receptor interaction and resulting downstream cellular cascades after MT and EGCG incubation, our study has demonstrated a different target of phenol action in T47D breast cancer cells: the PPARγ receptor. Clinical studies have identified PPARγ expression as a positive prognostic factor in ductal breast cancer [50] and higher concentrations of PPARγ seem to correlate inversely with grading, size and TNM stage of breast cancer [51, 52]. Until now, studies investigating a potential association between PPARγ and the anti-carcinogenic effect of MTE on breast cancer cells are lacking. Therefore, we investigated the PPARγ expression on protein and mRNA level after being exposed to matcha tea extract at concentrations of 5, 10, and 50 µg/ml). With the use of western blots, we could demonstrate a positive correlation between PPARγ expression and an increase in MTE concentration. Similar results emerged at the mRNA level, where an increased concentration of MTE leads to higher expression rates of PPARγ, but statistical significance was only reached at the highest MTE concentration.

As a nuclear receptor, the activity of PPARγ is depending on its intracellular localization and the activation through ligands, which can be natural agonists like fatty acids or prostanoids or synthetic ones like thiazolidinedione drugs [29]. Current data from in vitro and in vivo studies suggest an involvement of PPARγ in various cell signaling pathways. Via the stimulation of PPARγ ligands an upregulation of the expression of syndecan-1 and the activation of FAS ligand has been reported, which lead to an induction of cell apoptosis [53, 54]. Moreover, ligand activated PPARγ can interfere in the regulation of cell growth and cell cycle via the activation of promoter gene p53 and the upregulation of p53 and the target gene p21 [55]. This also triggers the intrinsic apoptotic pathway. Of special interest regarding a possible interference between PPARγ and the cell growth inhibitory effects induced by the compounds of matcha tea is the downregulation of the cyclin D1 promoter by PPARγ ligands. The reduced expression leads to the induction of a cell cycle arrest [56, 57]. Hong et al. could demonstrate that treatment of breast cancer cells with EGCG also leads to an inhibition of growth via downregulation of β-catenin, phosphorylated Akt and cyclin D1 [58]. With the dose dependent anti-carcinogenic effect and simultaneous upregulation of PPARγ, the influence of PPARγ via the downregulation of cyclin D1 seems plausible.

As our study seems to be one of the first investigating a potential involvement of PPARγ in the anti-carcinogenic effect of MTE in breast cancer, there is a lack of evidence regarding the alteration of cellular pathways. Moreover, as we analyzed an extract, the substrate responsible for the observed results still remains unknown. From other experiments of our group, we know that matcha tea also contains fatty acids, which could be responsible for PPARγ upregulation. In the future the experiment could be repeated with EGCG and fatty acids to evaluate their effects. In addition, decreased viability of T47D cells after MTE stimulation could be investigated in detail by immunohistochemistry or reverse transcriptase quantitative (RTQ)-PCR quantification of apoptosis-induced markers (for example, p53, p21, BCL2, Caspase 8/9). MTE incubation of estrogen receptor negative breast cancer cells (e.g. BT-20) could evaluate the effect of possible estrogen receptor interaction. Finally, our results may serve as a motivation for further investigations on matcha tea induced PPARγ alterations in cancer cell or animal models.

## Conclusions

In this in vitro study, we could demonstrate that incubation of T47D cells with different concentrations of MTE results in an increased expression of PPARγ and a dose dependent reduction of cellular viability. As PPARγ expression can result in anti-carcinogenic effects, more research in this field is needed to evaluate the observed results.

## Data Availability

The datasets used and analyzed during the study are available from the corresponding author on request.
